# Transcriptomic dataset from peripheral white blood cells of beef heifers at weaning

**DOI:** 10.1016/j.dib.2023.109046

**Published:** 2023-03-11

**Authors:** Priyanka Banerjee, Wellison J.S. Diniz, Soren P. Rodning, Paul W. Dyce

**Affiliations:** Department of Animal Sciences, Auburn University, Auburn, AL 36849, USA

**Keywords:** Beef heifers, Reproductive potential, Transcriptome, Weaning

## Abstract

Reproductive failure of replacement breeding animals is one of the leading causes of loss to the beef production industry. The losses are further increased due to the inability to diagnose the reproductive potential of the beef heifer prior to the breeding season until the pregnancy outcome. To overcome this problem, a system to discriminate beef heifers with varying reproductive potential as early and accurately as possible is demanded. The omics technologies, such as transcriptomics, could predict the future reproductive potential of beef heifers. Therefore, this manuscript provides the gene expression profile dataset using RNA-Seq identified from peripheral white blood cells (PWBC) of beef heifers at weaning. To accomplish this, the blood samples were collected at the time of weaning, processed to extract the PWBC pellet and stored at – 80 °C until further processing. After the breeding protocol (artificial insemination (AI) followed by natural bull service) and pregnancy diagnosis, the heifers that were pregnant to AI (*n* = 8) or remained open (n = 7) were utilized for this study. Total RNA was extracted from PWBC collected at the time of weaning from these samples and subjected to sequencing using the Illumina Nova-Seq platform. High-quality sequencing data was analyzed using a bioinformatic workflow based on FastQC and MultiQC for quality control, STAR for read alignment, and DESeq2 for differential expression analysis. Genes were considered significantly differentially expressed after adjustment with Bonferroni correction (*padj* ≤ 0.05) and absolute (log2 fold change) ≥ 0.5. Raw and processed RNA-Seq data were deposited and made publicly available on the gene expression omnibus database (GEO; GSE221903). To our knowledge, this is the first dataset investigating the change in the gene expression level as early as weaning to predict the future reproductive outcome in beef heifers. Interpretation of the main findings based on this data is reported in a research article titled “mRNA Signatures in Peripheral White Blood Cells Predicts Reproductive Potential in Beef Heifers at Weaning” [1].

**Specifications Table**

**Table:** 

Subject	Agricultural and Biological Sciences
Specific subject area	Animal Science, Omics: Transcriptomics
Type of data	RNA-Seq raw data (FASTQ format), .text file, .csv file and table
How the data were acquired	Total RNA was extracted from PWBCs and checked for quality and integrity. Libraries were prepared and sequencing was performed on the Nova-Seq Illumina platform. Paired-end 100 bp reads were generated for each sample.
Data format	Raw RNA-Seq data (FASTQ format), Raw read counts (.txt format), Normalized counts (.csv format)
Description of data collection	Blood samples were collected from beef heifers at weaning and processed to isolate PWBC pellets. Total RNA was extracted from PWBC pellets (n = 15) using Trizol reagent following standard procedures. After RNA quality check, the libraries were prepared, and sequencing was performed on the Nova-Seq platform at the Discovery life sciences (Hudson Alpha Institute of Biotechnology, Huntsville, AL, USA). Paired-end 100 bp reads were generated for each sample.
Data source location	Alabama Research and Extension Center (Auburn University) and Department of Animal Sciences, Auburn University, Auburn, Alabama, USA
Data accessibility	All relevant data (raw and processed RNA-Seq data) were deposited on: Repository name: Gene Expression Omnibus (GEO) Data identification number: GSE221903 Direct URL to data: https://www.ncbi.nlm.nih.gov/geo/query/acc.cgi?acc=GSE221903
Related research article	mRNA Signatures in Peripheral White Blood Cells Predicts Reproductive Potential in Beef Heifers at Weaning. Banerjee, P. B.; Diniz, W. J. S.; Hollingsworth, R.; Rodning, S. P.; Dyce, P. W. Genes (Basel). 14 (2023) 498. (https://doi.org/10.3390/genes14020498).

## Value of the Data

• This dataset provides the transcriptome profile of bovine PWBCs at weaning and allows a comparative RNA-Seq analysis between heifers of varying reproductive potential. The data could be used to predict the future reproductive outcome in heifers and understand subfertility in beef heifers.
• The data can be useful for researchers interested in studying genes and pathways underlying heifer fertility potential.
• The PWBC transcriptome profile at weaning provides the ability to determine the significant differentially expressed genes between the samples with varying future reproductive outcomes. This data could be used for a meta-analysis with a transcriptome profile generated at a different time point, such as during artificial insemination (AI). This will help to understand the role of potential candidate genes and altered pathways that may be contributing to a contrasting reproductive outcome.

## 1 Objective

Heifers are the backbone of the future cowherd. Significant efforts and resources are placed on the selection and development of heifers before the start of their breeding season. However, many heifers deemed reproductively mature, even after the phenotypic assessments, fail to conceive. To enhance reproductive efficiency, female replacement heifers with a high reproductive potential need to be selected. Identifying potential candidates at breeding can reflect reproductive health at the time of pregnancy initiation but will provide limited benefit to the producer, as significant costs have already been invested. Therefore, there is a high demand to develop a system (identify candidates) that can discriminate fertile beef heifers from infertile ones at an early stage of development, such as at weaning, when the replacement heifers are selected. This dataset was generated to unravel the gene expression profile from the PWBCs of beef heifers at weaning retrospectively classified as fertile (pregnant at AI) or subfertile (not-pregnant) based on the pregnancy status. The objective of generating and using this dataset is to identify potential candidate genes at weaning that could predict the future reproductive potential of beef heifers at an early age.

## 2 Data Description

The dataset in this article describes the whole transcriptome profile of PWBCs from beef heifers with varying reproductive outcomes (fertile = pregnant to AI and subfertile = not-pregnant). The raw reads were generated using the Nova-Seq platform (Illumina). Using a custom build bioinformatics pipeline, we performed data quality control, mapping and read counting, differential gene expression and functional analyses. We analyzed the data from 15 heifers grouped as pregnant to AI (*n* = 8) or not-pregnant (*n* = 7). The raw paired-end reads from 15 samples, the raw gene counts and the normalized counts are publicly available on the GEO database (GEO accession ID: GSE221903).

**Fig. 1:**
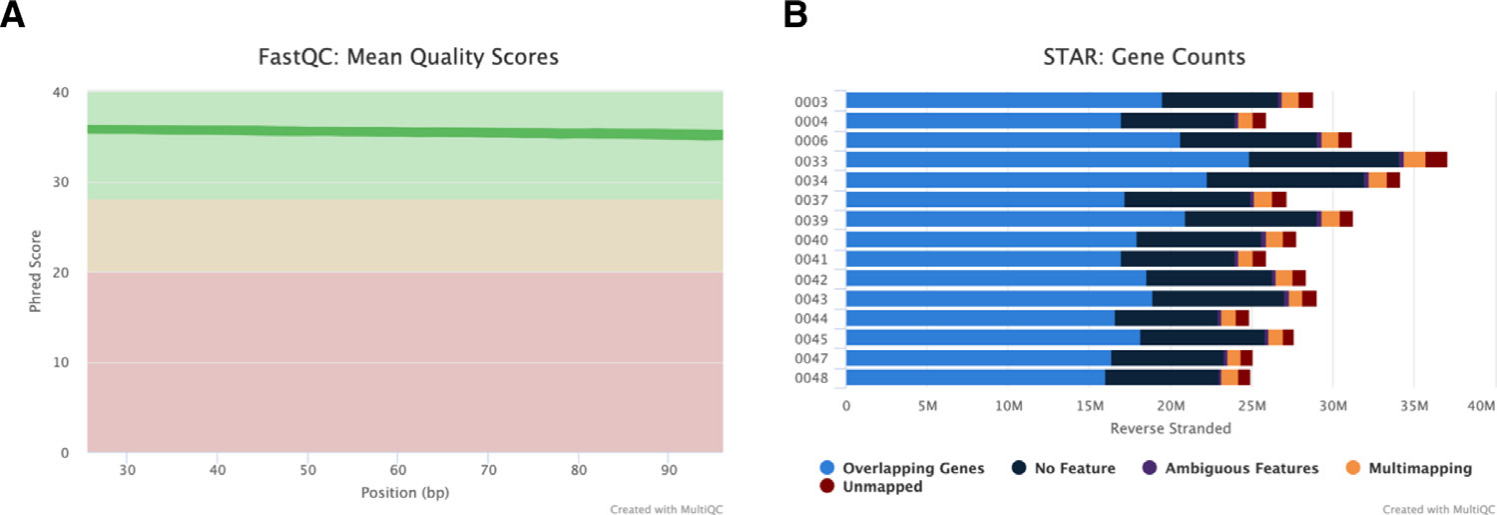
Overview of the RNA-Seq data from peripheral white blood cells (PWBC) of beef heifers at weaning and retrospectively classified as pregnant to AI or not-pregnant. (A) Overall Phred score of FASTQ files; (B) The statistical results from read mapping.

The sequencing generated, on average, 28.6 million reads per sample, of which 93.5% were uniquely mapped to the *Bos taurus* genome. Fig. 1A shows the read quality (Phred score was > 30, overall sequence read quality assessed by Phred score was > 30. Fig. 1B shows the statistics from results generated using *–quantMode GeneCounts* based on the mapping using the STAR aligner. The differential expression analysis between pregnant and non-pregnant heifers revealed a total of 92 significantly differentially expressed genes ((*padj* ≤ 0.05 and absolute (log2 fold change ≥ 0.5)).

Table 1 shows a summary of the metadata, sample description, mapping statistics per sample, and the experimental groups as previously described [1].

**Table 1:** RNA sequencing summary and mapping statistics.

Sample ID	M Seqs	M Aligned	% Aligned	Group
3	28.8	26.9	93.4	Not-pregnant
4	26	24.2	93.2	Pregnant to AI
6	31.2	29.4	94.1	Pregnant to AI
33	37.1	34.4	92.9	Not-pregnant
34	34.2	32.2	94.2	Pregnant to AI
37	27.2	25.2	92.6	Not-pregnant
39	31.3	29.3	93.7	Not-pregnant
40	27.8	25.9	93.3	Pregnant to AI
41	25.9	24.2	93.5	Pregnant to AI
42	28.4	26.5	93.4	Not-pregnant
43	29	27.3	94	Pregnant to AI
44	24.9	23.2	93	Not-pregnant
45	27.6	26.1	94.5	Pregnant to AI
47	25.1	23.6	94	Pregnant to AI
48	25	23.2	92.9	Not-pregnant

**Average**	**28.6**	**26.8**	**93.5**	

M Seqs = Million reads sequenced, M Aligned = Million reads aligned, % Aligned = Percentage of reads aligned to the reference genome, Group = Group to which the sample belongs according to the pregnancy status.

## 3 Experimental Design, Materials and Methods

### 3.1 Animal Handling

The Angus-Simmental crossbred heifers used in this study were developed as replacement heifers at the Alabama Research Center (Auburn University). At the time of weaning (∼238 days after birth), blood samples (10ml) were collected in EDTA-coated vacutainer tubes (Becton, Dickinson and Company, Franklin, NJ) and transported to the lab in ice for further processing. At breeding, all heifers followed the same breeding protocol, estrus synchronization and fixed-time artificial insemination (FTAI) as described previously [2]. Fourteen days following FTAI, the heifers were exposed to fertile bulls for 60 days.

### 3.2 Pregnancy Determination and Selection of Heifers

Pregnancy evaluation by transrectal palpation was performed by an experienced veterinarian. Depending on the presence or absence of conceptus at 75 days following AI, the heifers were classified as pregnant to AI, pregnant to natural service or non-pregnant. The heifers that became pregnant after AI and were not-pregnant were considered for this study.

### 3.3 Sample Collection and Processing

In the lab, the blood samples were centrifuged at 1,500g for 10 minutes at 4 °C. The buffy coat was separated and added into a fresh centrifuge tube with 14ml red blood cell lysis buffer (0.15 M ammonium chloride, 10 mM potassium bicarbonate, 0.1 mM EDTA, Cold Spring Harbor Protocols) and incubated for 10 minutes at room temperature. The tubes were centrifuged at 500g for 5 minutes at 4 °C to pellet the PWBCs. The supernatant was discarded, and the pellet was re-suspended in 700 μl of PBS/ 2% FBS and centrifuged at 500g for 5 minutes. The supernatant was discarded, and the clean PWBC pellet was stored at – 80 °C until further processing.

### 3.4 RNA Extraction, Library Preparation and Sequencing

Total RNA was extracted from the PWBC of 15 samples collected at the time of weaning. The total RNA was extracted using Trizol reagent (Invitrogen, Carlsbad, CA, USA) following standard procedures. RNA purification and DNase digestion were done using an RNA clean and concentrator kit (Zymo Research, Irvine, CA, USA). The quality and RNA integrity of total RNA was assessed using Agilent Bioanalyzer with the Agilent RNA 6000 Nano kit (Agilent, Santa Clara, CA, USA). The libraries were prepared, and sequencing was performed on the Nova-Seq platform at the Discovery life sciences (Hudson Alpha Institute of Biotechnology, Huntsville, AL, USA). Paired-end 100 bp reads were generated for each sample.

### 3.5 Data Analysis

After sequencing, the quality control of raw data was performed using FastQC v0.11.9 [3] and MultiQC v1.12 [4]. The reads were evaluated based on average read length, adapter content, per sequence GC content, and sequence quality scores. The raw reads were mapped using STAR aligner v2.7.5 using Ensemble's *Bos taurus* reference genome (ARS UCD1.2). The sequencing from all the samples yielded an average of 28.6 million reads per sample, of which 93.5% were uniquely mapped to the *Bos taurus* genome (Table 1). The post-mapping quality control of the reads was done using MultiQC v1.12. The read quantification was performed using a STAR aligner [5] to obtain raw counts per gene. The read counts were transformed to counts per million (CPM) using edgeR v3.28.1 [6]. Genes with CPM < 1 in 50% of the samples were filtered out. The filtered raw gene counts were then subjected to differential expression analysis using DESeq2 v1.26.0 [7]. The pregnancy status (pregnant or non-pregnant) was considered for the design model used on the DESeq2 R-package. The differentially expressed genes with *padj* ≤ 0.05 and absolute (log2 fold change) ≥ 0.5 were considered significant.

## Ethics Statements

All procedures involving animals were approved by Institutional Animal Care and Use Committee (IACUC) at Auburn University and the guide for the Care and Use of Laboratory Animals (IACUC protocol number 2015-2786 and 2019-3591).

## CRediT authorship contribution statement

**Priyanka Banerjee:** Conceptualization, Methodology, Software, Data curation, Writing – original draft, Software, Validation, Writing – review & editing. **Wellison J.S. Diniz:** Methodology, Writing – review & editing. **Soren P. Rodning:** Methodology, Writing – review & editing. **Paul W. Dyce:** Conceptualization, Methodology, Supervision, Funding acquisition, Writing – review & editing.

## Declaration of Competing Interest

The authors declare that they have no known competing financial interests or personal relationships that could have appeared to influence the work reported in this paper.

## Data Availability

Transcriptome profiling from peripheral white blood cells in fertile and subfertile beef heifers at weaning (Original data) (NCBI).
